# Developing global guidance on human milk banking

**DOI:** 10.2471/BLT.21.286943

**Published:** 2021-10-20

**Authors:** Mirriam Tyebally Fang, Efstratios Chatzixiros, Laurence Grummer-Strawn, Cyril Engmann, Kiersten Israel-Ballard, Kimberly Mansen, Deborah L O'Connor, Sharon Unger, Marisa Herson, Gillian Weaver, Nikola Biller-Andorno

**Affiliations:** a Institute of Biomedical Ethics and History of Medicine, University of Zurich, Winterthurerstrasse 30, 8006 Zurich, Switzerland.; b Department of Health Product Policy and Standards, World Health Organization, Geneva, Switzerland.; c Department of Nutrition and Food Safety, World Health Organization, Geneva, Switzerland.; d PATH, Seattle, United States of America.; e Department of Nutritional Sciences, University of Toronto, Toronto, Canada.; f Sinai Health, Toronto, Canada.; g School of Medicine, Deakin University, Geelong, Australia.; h Human Milk Foundation, Harpenden, England.

## Abstract

Donor human milk is recommended by the World Health Organization both for its advantageous nutritional and biological properties when mother’s own milk is not available and for its recognized support for lactation and breastfeeding when used appropriately. An increasing number of human milk banks are being established around the world, especially in low- and middle-income countries, to facilitate the collection, processing and distribution of donor human milk. In contrast to other medical products of human origin, however, there are no minimum quality, safety and ethical standards for donor human milk and no coordinating global body to inform national policies. We present the key issues impeding progress in human milk banking, including the lack of clear definitions or registries of products; issues around regulation, quality and safety; and ethical concerns about commercialization and potential exploitation of women. Recognizing that progress in human milk banking is limited by a lack of comparable evidence, we recommend further research in this field to fill the knowledge gaps and provide evidence-based guidance. We also highlight the need for optimal support for mothers to provide their own breastmilk and establish breastfeeding as soon as and wherever possible after birth.

## Résumé

Lorsque la mère est dans l'impossibilité d'allaiter, l'Organisation mondiale de la Santé recommande d'opter pour le lait humain provenant de donneuses, tant pour ses propriétés nutritionnelles et biologiques que pour la contribution avérée qu'il apporte à la lactation et à l'allaitement quand il est utilisé à bon escient. Un nombre croissant de banques de lait humain s'établissent dans le monde entier, en particulier dans les pays à faible et moyen revenu, afin de faciliter la collecte, le traitement et la distribution de lait humain provenant de donneuses. Cependant, contrairement à d'autres produits médicaux d'origine humaine, il n'existe aucune norme minimale de qualité, de sécurité et d'éthique en la matière, et aucun organe de coordination global n'a été créé pour guider les politiques nationales. Dans le présent document, nous évoquons les principaux obstacles à la progression des banques de lait humain, notamment l'absence de définition claire ou de registre de produits; les problèmes relatifs à la réglementation, la qualité et la sécurité; ainsi que les questions éthiques entourant la commercialisation et l'exploitation potentielle des femmes. Jugeant cette progression limitée par le manque de données comparables, nous encourageons à mener d'autres recherches dans ce domaine pour combler les lacunes et fournir des orientations fondées sur des preuves. Nous soulignons également la nécessité d'offrir un soutien optimal aux mères afin qu'elles puissent produire leur propre lait et allaiter autant que possible immédiatement après la naissance.

## Resumen

La Organización Mundial de la Salud recomienda la leche humana donada tanto por sus ventajosas propiedades nutricionales y biológicas cuando no se dispone de la propia leche materna como por su reconocido apoyo a la lactancia y al amamantamiento cuando se utiliza de manera adecuada. Cada vez se crean más bancos de leche humana en todo el mundo, sobre todo en los países de ingresos bajos y medios, para facilitar la recogida, el procesamiento y la distribución de leche humana donada. Sin embargo, a diferencia de lo que ocurre con otros productos médicos de origen humano, no existen estándares mínimos de calidad, seguridad y ética para la leche humana donada ni un organismo mundial de coordinación que sirva de base a las políticas nacionales. En este documento se exponen los principales problemas que impiden el progreso de los bancos de leche humana, como la falta de definiciones claras o de registros de productos; los problemas relacionados con la regulación, la calidad y la seguridad; y las preocupaciones éticas sobre la comercialización y la posible explotación de las mujeres. Dado que el progreso de los bancos de leche humana se ve limitado por la falta de evidencias comparables, se recomienda seguir investigando en este campo para compensar los vacíos de conocimiento y proporcionar una guía asistencial. Asimismo, se destaca la necesidad de apoyar al máximo a las madres para que se provean de su propia leche materna y establezcan la lactancia materna tan pronto y siempre que sea posible después del nacimiento.

## ملخص

توصي منظمة الصحة العالمية بلبن الأمهات من الأطراف المتبرعة به، وذلك لخصائصه التغذوية والبيولوجية المفيدة عندما لا يتوفر لبن الأم، ولدعمه المعترف به للرضاعة والرضاعة الطبيعية عند استخدامه بشكل مناسب. يتم الآن إنشاء عدد متزايد من بنوك لبن الأمهات في جميع أنحاء العالم، وخاصة في الدول ذات الدخل المنخفض والدخل المتوسط، لتسهيل جمع ومعالجة وتوزيع لبن الأمهات من الأطراف المتبرعة به. ومع ذلك، وعلى النقيض من المنتجات الطبية الأخرى من أصل بشري، ليس هناك حد أدنى للجودة والسلامة والمعايير الأخلاقية للبن الأمهات من الأطراف المتبرعة به، ولا توجد هيئة عالمية منسقة لتوجيه السياسات الوطنية. نحن نقدم القضايا الرئيسية التي تعوق التقدم في بنوك لبن الأمهات، بما في ذلك الافتقار إلى تعريفات أو سجلات واضحة للمنتجات؛ مثل المسائل المتعلقة بالتنظيم والجودة والسلامة؛ والمخاوف الأخلاقية المتعلقة بالجانب التجاري والاستغلال المحتمل للمرأة. مع إدراك أن التقدم في بنوك حليب الأمهات محدود بسبب الافتقار إلى أدلة قابلة للمقارنة، فإننا نوصي بإجراء مزيد من البحث في هذا المجال لسد الفجوات المعرفية وتقديم إرشادات قائمة على الأدلة. ونحن نسلط الضوء أيضًا على الحاجة إلى الدعم الأمثل للأمهات ليقمن بتقديم ألبانهن، وبدء الرضاعة الطبيعية بأسرع ما يمكن وحيثما أمكن بعد الولادة.

## 摘要

世界卫生组织建议人们捐赠母乳，因为当母亲自身的母乳不可用时，捐赠母乳具有充足的营养价值和生物特性，若使用得当，它可以对哺乳和母乳喂养提供有利的支持。世界各地正在建立越来越多的母乳库，特别是在中低收入国家，以推进捐赠母乳的收集、加工和分配。但是，与其他人类制造的医疗产品相比，捐赠母乳没有最低质量、安全和道德标准，也没有全球协调机构为国家政策提供信息。我们提出了阻碍母乳储存进展的关键问题，包括缺乏明确的产品定义或注册信息；监管、质量和安全方面的问题；以及对妇女商业化和潜在剥削的道德问题。认识到母乳储存的进展因缺乏可比证据而受到限制，我们建议在该领域展开进一步研究以填补知识空白并提供循证指导。我们还强调需要为母亲提供最佳支持，以支持她们提供自己的母乳，在孩子出生后尽快并尽可能地进行母乳喂养。

## Резюме

В случае отсутствия материнского молока Всемирная организация здравоохранения рекомендует использование донорского грудного молока как из-за его полезных питательных и биологических свойств, так и из-за общепризнанной поддержки лактации и грудного вскармливания при правильном использовании. Во всем мире создается все больше банков грудного молока, особенно в странах с низким и средним уровнем доходов, для облегчения сбора, обработки и распределения донорского грудного молока. Однако, в отличие от другой медицинской продукции человеческого происхождения, для донорского грудного молока не существует минимальных стандартов качества, безопасности и этики, а также отсутствует координационный глобальный орган для разработки национальной политики. Авторы раскрыли ключевые проблемы, препятствующие прогрессу в создании банков грудного молока (включая отсутствие четких определений или реестров продуктов), вопросы регулирования, качества и безопасности, а также этические проблемы в связи с коммерциализацией и потенциальной эксплуатацией женщин. Признавая, что прогресс в создании банков грудного молока ограничен отсутствием сопоставимых данных, авторы рекомендуют продолжить исследования в этой области, чтобы заполнить пробелы в знаниях и предоставить рекомендации, основанные на фактических данных. Подчеркивается также необходимость оптимальной поддержки матерей, чтобы они сами кормили ребенка грудью и по мере возможности начинали грудное вскармливание как можно скорее после родов, где бы они ни находились.

## Introduction

The World Health Organization (WHO) recommends donor human milk for low- and very-low-birthweight infants, as well as small and sick newborns who cannot be fed their mother’s own milk.^1^^,^^2^ Human milk banks organize the collection, processing and distribution of donor human milk, acting as a bridge between donors and recipients, and aiming to ensure the quality and safety of this biological product. These services should be integrated components of care for small and sick newborns and a core mechanism for supporting lactation and breastfeeding.^3^^,^^4^

The number of human milk banks around the world that aim to improve neonatal health through the provision of donor human milk is increasing. In 2020, it was estimated that there were 756 milk banks in 66 countries,^5^ with an increasing number of milk banks being established in low- and middle-income countries. However, there is no global coordinating body issuing minimum quality, safety and ethical standards to inform national policies on donor human milk. While milk banks may need to adapt to the restrictions, resources and needs of their local area, they need to apply similar baseline processes to provide safe and high-quality donor milk.^6^

Experts in human milk banking have suggested that the subject would benefit from global guidance on a variety of important issues, and have called for international leadership and local government oversight.^5^^,^^7^^,^^8^ We discuss here the key issues in human milk banking that need to be addressed by up-to-date, evidence-based global guidance, as well as the knowledge gaps currently limiting this process. We also present case studies demonstrating how human milk-banking systems can be implemented, keeping in mind support for lactating mothers, and integrating donor human milk practices with other interventions for small and sick babies.

## Definitions

Standardizing terminology is the first step for comparing data related to donor human milk and allowing a critical appraisal of practices related to human milk banking. Any exchange of data should as a minimum clearly state the relevant characteristics of the donor pool;^9^ the process of screening the donor and the microbial content of the milk;^10^^,^^11^ the procedures for processing and storage of the milk;^12^ and the types and amounts of fortification, if used.^13^^,^^14^ In Box 1 we present some key definitions that need to be harmonized in the literature on human milk banking to support progress in the field.

Box 1 **Definitions of terms used for human milk banking**

**Human milk bank**

A service established to (i) recruit and screen human milk donors, (ii) collect donated human milk, (iii) screen, process and store donor human milk, and (iv) distribute this milk to meet an infant’s specific needs for optimal health.^6^ Ideally, the service should be linked with support for breastfeeding mothers.^3^^,^^4^

**Mother’s own milk**

Human milk from an infant’s own mother.^6^

**Donor human milk**

Human breastmilk donated by a mother and provided voluntarily to a human milk bank to be fed to an infant other than her own.^10^ Stating if donor human milk is pooled, raw, pasteurized or fortified is important:

• Pooled milk is donated milk from a single donor mother or from several donors at various stages of lactation. The pooling of donations aims to homogenize the components of the milk.^15^

• Raw milk is unprocessed donated milk. The intention is to retain as many of the active and advantageous biological and microbial components within the milk.^16^

• Pasteurized milk is processed to reduce the microbial load. Different pasteurization procedures have different effects on bacterial loads, nutritional components and bioactive components.^17^^,^^18^ Possible pasteurization procedures include: holder pasteurization;^17^ retort processing;^19^ high-temperature short-time processing;^18^ high hydrostatic pressure processing;^18^^,^^20^ and ultraviolet-C irradiation.^18^

• Fortification is the addition of nutrient fortifiers to donor human milk.^13^^,^^21^ Types of fortifiers include: concentrated bovine-based protein powders;^21^ liquid concentrates;^21^human milk-based concentrates;^21^ and cow’s milk formula powders.^21^ More evidence is needed to understand both the benefits and potential harms of fortification on infant health and development.^14^

**Direct breastfeeding**

Infants who directly receive their mother’s own milk by suckling at the mother’s breast.

**Expressed feeds**

Infants may also be fed their mother’s own milk via other means, such as a tube, cup or bottle. Such milk may have been expressed by hand or via a breast pump and stored, either at home in a refrigerator or freezer or at a human milk banking facility.^22^

## Regulatory issues

Despite the growth in human milk banking, the majority of human milk banks are not routinely being regulated at the national level. Global regulation is lacking altogether. Robust national regulation would ensure that the operators of human milk banks are accountable for minimum quality and safety standards; that human milk banks are sustainable as part of the wider health-care system supporting both neonatal and maternal health; and that the operation of human milk banks does not undermine breastfeeding. 

As a start, current and future human milk banks should be registered with an appropriate national regulatory authority. The criteria for registration should therefore be further defined. For example, a hospital may need to be a certified mother- and baby-friendly hospital,^23^ or an independent milk bank may need to be integrated into the wider health-care system. Countries such as Viet Nam have developed prerequisites for the establishment of human milk banks that are aligned with national hospital quality standards.^24^^,^^25^ There is a need for policy recommendations that facilitate the appropriate use of donor human milk and that ensure donor milk practices are integrated with other interventions for small and sick babies such as skin-to-skin care. A national regulatory authority would be responsible for enforcing compliance with quality and safety standards as a prerequisite for registration of a milk bank and as a condition for its continuing operation. Establishing safeguards against conflicts of interest in the funding of human milk banks would be beneficial as well as guidelines that facilitated sustainable practices, equity of access to donor milk and an ethical code of conduct for human milk banks. Brazil provides an example of an established model of human milk banking for low- and middle-income countries which successfully integrates human milk banks into the larger health-care system including the use of innovative, low-cost methods (Box 2).

Box 2 **Case study: a nationalized, integrated human milk-bank programme, Brazil**

Brazil has successfully demonstrated the effectiveness of a nationalized, integrated human milk-banking programme that includes breastfeeding promotion, lactation support and the provision of donor milk. The milk is provided from the Global Network of Human Milk Banks (*Rede Global de Bancos de Leite Humano*), and the expanded Ibero-American Network of Human Milk Banks (*Programa Iberoamericano de Bancos de Leche Humana*). The model in Brazil is considered to be a leading global model of human milk banking, operating more than 224 human milk banks or houses of lactation, with more than 214 collection points in the country, and with outreach activities extending to more than 25 countries.^26^ From 2000–2019, the system distributed over 2 815 420 litres of donor human milk, collected from over 2 466 160 donors.^27^

The simplified systems used in Brazil facilitate sustainable operations and act as a model for low- and middle-income countries. The system includes collecting donated milk in glass mayonnaise jars and manual pasteurization rather than expensive automated pasteurizers. To protect, promote and support breastfeeding and lactating mothers, Brazil has integrated mother and baby centres that serve to both provide lactation support and act as a collection station for human milk donations. In-service training is provided for health-care staff. Support for the individual mother and her infant is provided through professional lactation counselling, kangaroo mother care, rooming-in, mother-to-mother support and peer-support network-building. These services are integrated into and reinforce the human milk-banking system, with the ultimate goal of ensuring all infants have equitable access to human milk. Mothers are informed and provided with breastfeeding guidance and resources both antenatally and postnatally to support their breastfeeding practices. Mothers are also educated on the options available to them when they have milk in excess of their infant’s needs.

The appropriate classification of human milk is another fundamental issue that needs to be addressed by regulatory authorities, as it affects the legal frameworks governing the handling and use of the milk. Although human milk is most commonly classified as a food or nutritional therapy,^6^ it is in essence a biological material derived from the human body and processed with the intention of clinical application. WHO therefore considers donor human milk a medical product of human origin.^28^ A consensus on the nature of human milk as a regulated substance needs to be agreed upon before discussions can begin on a unified set of procedures on safe handling and precautions for the appropriate use of the milk. Additional areas that may require policies and regulation include other uses of human milk, such as an adjunct to cancer therapy,^29^^–^^31^ the import and export of donor human milk products between countries, and preventing exploitation of women by commercial milk banks.

## Quality criteria

An important aim in developing guidance for human milk banking is ensuring that milk banks in different settings attain a minimum level of quality and safety. Guidance documents will need to provide recommendations on how to attain these standards.^32^ Quality and safety criteria should cover how the milk bank is operated: the recruitment of donors, the quality of the end product and the appropriate use and distribution of the donor milk. A challenge for human milk banks is how to develop evidence-based criteria to guide such standards when scientific evidence is lacking on the desired quality and safety of donor human milk.

The aim of quality-control processes in human milk banking would be to optimize the protection of the nutrient content and biological activity of the milk. Some processes, especially pasteurization, alter the composition of human milk and diminish its bioactive components.^17^^,^^18^ One limiting step in optimizing the composition of the milk, however, is the inability to reliably analyse and hence characterize both raw and processed donor human milk. Most milk banks do not have access to the highly specialized laboratories required to accurately measure the macronutrient and immunological components of human milk. Milk banks may even employ commercially available technical equipment developed for the dairy industry. The relevance of any analyses is uniquely difficult to assess for human milk because the activity of bioactive components in the milk and their contribution to clinical outcomes depend on interactions with the infant gut.

In the absence of a clearly defined standard of quality, optimal donor human milk could provisionally be defined as “human milk that is most similar to that of the mother’s own milk for a particular infant profile, with the highest retention of beneficial properties and the lowest pathogenic activity.” Given the inherent variations in donated human milk, and rather than a single standardized product, donor human milk could be conceived as being a range of donated human milks sourced and processed in different ways to meet the needs of clearly defined end-users. Such users could include preterm infants, infants with specific underlying health conditions, or well infants without access to sufficient (or any) mother’s own milk. Therefore, donor human milk banking would involve matching the most appropriate donor milk to a particular infant at the right time and conditions and under appropriate guidelines to prevent overuse of the milk.^33^ Optimizing donor human milk would be about making it effective for a specific scenario. Underpinning the use of donor human milk at all times would be using it to support the recipient mother in establishing lactation and the successful breastfeeding of her own infant wherever possible.

Any actions to mitigate the potential risk of microbial transmission or contamination from donor human milk need to be balanced against the decontamination processes which affect the beneficial natural microbiome present in human milk. As with other medical products of human origin, a risk-based approach is needed in determining acceptable safety thresholds, with human milk banks adhering to minimum required standards. Factors affecting safety include inclusion criteria for donors, screening of donors or milk samples for infectious diseases, determining the need for pasteurization, microbial testing of milk, and the use of handling and storage protocols that prevent microbial contamination. An analysis and validation of current practices in microbial testing would also be useful, as there is presently a large variation in methods and samples used across human milk banks. Milk banks would benefit from guidance on the indications for pathogen testing, types of pathogens to test for, optimal testing methods and their alternatives, acceptable limits of test results, and any further actions required.

## Quality management

The handling of donor human milk needs to adhere to the same principles applied to other medical products of human origin,^28^ regardless of how donor human milk is classified by a given country. The fundamental principles for a human milk quality management system are risk containment and mitigation strategies; surveillance of processes related to human milk banks; traceability of distributed donor human milk; and adverse events response. Further quality management principles that would be universally applicable can be derived by developing a clear hazard profile of donor human milk. Hazard analysis critical control point is an example of a management system used by some milk banks. The system addresses potential safety issues through the analysis and control of biological, chemical and physical hazards, from raw material production, procurement and handling, to manufacturing, distribution and consumption of the finished product.^4^^,^^11^^,^^34^ Using such a system, human milk banks would have some scope to determine the processes they will apply to the hazard identified, such that the risk to the intended recipient is deemed acceptable to the population. This method respects the differences in milk-banking practices among countries, while still outlining the essential quality standards that need to be met. Lastly, human milk banks should have a quality assurance programme involving both internal and external audits that validate and verify that the milk bank is operating according to the decreed standards and that expected outcomes are being met.

Box 3 outlines the human milk-banking system in Kenya which, unlike that of Brazil, is relatively new. Stakeholders and technical experts, both local and foreign, were involved in determining the need for human milk banking, and subsequently initiating sustainable locally appropriate implementation strategies. Continuing evaluations have facilitated quality improvements, while an impact and feasibility analysis was conducted before considering expanding human milk banking in the region.

Box 3 **Case study: the mother–baby friendly initiative plus programme, Kenya**

With the exception of South Africa, human milk-banking systems are largely absent in Africa. Recognizing the urgent need to reduce neonatal mortality, the Kenyan government and Kenyan policy-makers sought to improve access to human milk for high-risk, small and sick newborns. In 2016, a national technical committee comprising experts in various relevant fields was established to guide locally appropriate implementation strategies.

The aim was to establish a mother–baby friendly initiative plus model^6^^,^^35^ of human milk banking. This model reflects a comprehensive approach to strengthening access to human milk for all infants through specialized lactation support for mothers of small and sick newborns as well as through the provision of safe donor human milk from a human milk bank when needed. To ensure the sustainability of the initiative, a validated, phased approach^25^^,^^35^ was employed, led by the Kenyan health ministry.

The first phase involved formative research to determine the acceptability of the initiative^36^ and facility readiness. Learning exchange programmes with South Africa and Scotland, United Kingdom of Great Britain and Northern Ireland, helped build local technical competence. Intensive lactation training ensured staff were equipped to support breastfeeding mothers and use donor human milk appropriately. Locally specific Kenyan guidelines on human milk banking were developed, with standard operating procedures established based on hazard analysis critical control point quality assurance training. The second phase began in March 2019 with the launch of the mother–baby friendly initiative plus programme. The programme was intensively mentored by the nongovernmental organization PATH and other technical experts on human milk banks, with a strong focus on quality improvement. In the third phase, the African Population Health and Research Center evaluated the programme including its impact and long-term feasibility. The success of the programme can be attributed to the systematic, phased approach in creating a locally appropriate model, as well as the joint leadership among child health and nutrition teams at the national and county levels. These factors have been important in demonstrating scalability of the programme in Kenya and beyond.

## Data registries

Maintaining a registry would allow managers of human milk banks to understand the factors that contribute to improvements in technical and medical practice, such as donation trends, supply and demand, and the medical resources available in different countries. Depending on the breadth of data collected, a registry may be a biovigilance tool to facilitate evaluation of the outcomes of a donor milk system.

Determining the purpose of data captured in a registry is important and would enable human milk banks to be integrated into the wider health-care system. Registries could consider including data on lactation management, neonatal feeding indicators and population-based data (such as the demographic characteristics of pregnant women, the total number of pregnant women, maternal health, and type of delivery), together with adverse events and clinical outcomes. Other categories of data might include human milk-banking processes and systems, and the distribution and use of the milk. 

Maintaining a registry may also be a practical strategy to build a solid research database in human milk banking. The data collected could help bridge current knowledge gaps, including clarifying the different quality and safety characteristics of donor human milk and the milk’s suitability for various infant needs. Such data could also help to identify whether systems for supporting mother’s own milk need to be further strengthened alongside these efforts towards improved human milk banking.

To facilitate biovigilance, donor human milk needs to be uniquely identified so that it can be traced from each donor to each recipient. The traceability of donor human milk has been improved by its inclusion in the International Society of Blood Transfusion 128 unique product coding system.^37^ Nevertheless, pooling practices in milk banks continue to pose a challenge for traceability, as milk from several women can be pooled and distributed to a large number of neonates. Given the challenge in traceability, human milk banks – particularly those operating at the regional or national level – would benefit from guidance on the difficult task of recalling a product. As a minimum, adverse events should be reported to professional associations as well as regulatory authorities that are responsible for following up adverse events and implementing measures to investigate and respond to these events. Those involved in human milk banking may also want to consider creating a global outcome registry and a standardized global reporting process for reporting adverse events from donor human milk. WHO currently supports two global databases related to medical products of human origin usage: the Global Observatory on Donation and Transplantation^38^ and the Notify Library on vigilance and surveillance of adverse events.^39^ To establish a global data set for donor human milk, human milk banks would first need to agree on harmonized operational definitions and to establish national registries. A global outcome registry could facilitate the effective identification and resolution of issues related to human milk banking.

## Support for mothers

Concerns exist that the availability of donor human milk and support for milk banks may divert support from assisting mothers with establishing and maintaining their own milk production.^3^ The use of donor human milk may be seen as more convenient and less time-consuming compared with supporting breastfeeding mothers.^40^ Alternatively, a mother may want to feed her baby human milk but may not want to breastfeed herself by way of preference. In this way, donor human milk may start replacing mother’s own milk. While donor human milk may be more convenient, it should not be considered an equivalent replacement.

There is currently no international guidance on the ideal clinical pathway for determining the appropriate use of donor human milk or for implementing systems to prevent potential overuse and misuse of the milk. One suggestion is situating milk banking in the context of improving access to mother’s own milk, such that it supports breastfeeding and lactation outcomes rather than supersedes them. Inadequacies in breastfeeding and lactation support could be addressed alongside the need to supply donor human milk, with the priority being mother’s own milk. There are strong existing models as examples for other health systems to learn from and adapt. These initiatives have introduced human milk banking into their existing breastfeeding protection, promotion and support efforts, reaching far more infants with human milk as a whole than with donor milk alone.^41^

## Ethical issues

An important role for any guidance on human milk banking and donor human milk is mitigating ethical concerns. Chief among these concerns are the adequacy of support for donor mothers who may be at risk of exploitation, the commercialization of donor human milk, and ensuring equal opportunities to both provide and gain access to donor human milk.^8^ Governing institutions could consider how to monitor the potential exploitation of human milk donors.^42^

While donors should be fairly reimbursed for the significant commitment of regular pumping of breastmilk for a period of time, which could last for years, the provision of financial incentives may lead to adverse outcomes. Poorer women may be motivated to provide milk at the expense of their own health, placing themselves and their infants at risk. Financial rewards may encourage donors to conceal unsafe personal practices and medical history. These risks may be exacerbated by the growth of commercial use of human milk by for-profit companies, which is affecting neonatal feeding practices in both high- and low-income countries.^43^ Additionally, the position of donor human milk within the International Code of Marketing of Breast-milk Substitutes as well as the protections that countries should consider needs clarification. Scrutiny is also needed for the commercial use of human milk for purposes apart from infant health, such as bodybuilding supplements^44^ and beauty products,^45^ that might increase demand for donor human milk. Informed consent should be obtained from donors as well as recipients, with clear descriptions of how donor human milk is used and the potential risks and benefits, including the possible consequences for the mother and her infant.

Access to donor human milk tends to be inequitable both within and between countries, regions and even towns and cities. Factors that work against the fair distribution of human milk include commercial practices, lack of investment in non-profit human milk-banking facilities and the lack of insurance coverage to reimburse users of donor human milk. To ensure fair access to donor human milk across social classes, we need policies and appropriate governance to understand the demand, ensure sufficient supplies and provide financial support if needed. Human milk banking in India shows the benefit of involving national regulatory authorities in ensuring not just the sustainability, safety and quality of donor human milk, but also fair access to donor human milk across socioeconomic levels (Box 4).

Box 4 **Case study: comprehensive lactation management systems, India**

The first human milk bank in India was established in 1989, in Lokmanya Tilak municipal medical college and general hospital in Mumbai. Although initial growth was slow, 22 human milk banks were subsequently established between 2005 and 2015. As of 2021, there are over 90 human milk banks in India.^46^ The rapid growth in human milk banking recently is attributed to increased government support at both the national and state level. Previously, most human milk banks were established and run by nongovernmental organizations. With one of the highest burdens of neonatal mortality in the world, the Indian government has prioritized access to human milk as a key strategy in reducing neonatal mortality, with financial support for human milk banking provided at the state level through hospital funds.

The national guidelines on lactation management centres in public health facilities^34^ were drafted by a local technical expert group in response to the need for guidance and operational procedures specific to the local Indian context. India’s comprehensive lactation management centres were based on the Brazilian model and the mother–baby friendly initiative plus approach.^35^ Priority is given to universal access to human milk, with a focus on supporting a mother’s own lactation and supplementing it with safe donor human milk only when required. The rapid increase in human milk banking in India has been further facilitated by the milk banks’ ability to innovate and adapt human milk banking processes to meet local resource constraints. Solutions include the development of a locally manufactured, low-cost pasteurizer; the use of easily accessible metal containers to store donated human milk; and creative staffing models involving part-time technicians. The Human Milk Banking Association of India has since been established as a regional network for advancing evidence-based practices and advocacy for human milk banking in India.

The issue of milk kinship in Muslim communities also remains a barrier to equitable access to donor human milk globally.^47^ Despite the emergence of milk banks in a few Muslim-majority countries,^48^ this sociocultural factor requires special focus at the global and national levels and draws attention to the need to embed the ethical values of the community of donors and recipients in national legislation and human milk-banking policies.

## Global coordination

Human milk banking would benefit from a unified global coordination body with global representatives from various relevant technical backgrounds, including experts in child and newborn health and biosafety. The range of responsibilities of this global body would include advocacy work; engaging with policy development and implementation; quality management audits; overseeing data-sharing among milk banks including milk-banking registries; providing direction on safe functioning of milk banks during periods of crisis;^5^^,^^49^^,^^50^ and mentoring for both existing and newer milk banks. Crises such as natural disasters and pandemics present a challenge to maintaining safe human milk supplies, with local issues amplified by the absence of globally agreed operational safety guidelines.^5^^,^^49^^,^^50^ The challenges are exacerbated by the absence of a formal global mechanism for rapid communication among milk banks, and the lack of data and infrastructure to ensure responsiveness of the milk-banking system.^5^^,^^49^^,^^50^ Human milk-banking systems need strengthening to ensure that the safe provision of donor milk remains an essential component of early and essential newborn care during routine care or emergencies worldwide.^5^^,^^49^^,^^50^

## Conclusion

The ability to provide human milk to all infants who need it reflects a country’s ability to achieve important health and development goals, improve neonatal health, adhere to minimum standards of quality and safety, and develop robust quality management systems. Realizing these goals while navigating important ethical concerns requires guidance from both national and global leadership and regulatory bodies. Global guidance is needed on the standard operating procedures that are required for the operation of human milk banks, considering the different contexts in which milk banks operate internationally. In Box 5 we summarize the key points raised above and suggest recommendations to advance the field of human milk banking. An in-depth review of the existing evidence on human milk banking and highlighted knowledge gaps will be an important next step towards evaluating the potential role of WHO guidelines on the subject.

Box 5 **Recommendations on the way forward for human milk banking**

**Regulatory issues**

- Establish global guidance for governments to determine the most suitable level of regulatory oversight to increase accessibility to human milk, while ensuring the highest level of quality and safety. The classification of donor human milk needs to be determined, keeping in mind its human origins and the existing regulatory framework around medical products of human origin. Classification carries implications for accessibility, cost, payment and insurance coverage, regulatory oversight and protections of the donor, the recipient and the product.
- Assess existing classification models for human milk (for example, as a food; as a nutrition therapy; as a medical product of human origin; or an undefined category). The aim would be to balance the advantages and disadvantages of that classification type in the local setting and align with local regulatory frameworks.
- Ensure that national regulatory bodies and maternal, newborn and nutrition stakeholders collaborate to assess the appropriate mechanisms in their local settings. Stakeholders could include the local professional associations related to maternal and newborn health and nutrition, as well as representatives from potential donor and recipient mothers. The classification of donor human milk and regulation of human milk banking need to align with local frameworks developed to strengthen maternal and newborn care and nutrition.

**Quality and safety of donor human milk**

- Establish global standards on minimum requirements of safety and quality with guidance addressing all aspects of human milk banking (screening, donation, storage, pooling, pasteurization and processing, microbial screening and allocation of donor human milk).
- Ensure that global standards on human milk are in line with shared goals among milk banks worldwide. These goals need to be feasible, appropriate and adaptable to address the diversity of culture, disease risk, government oversight, resources, and health-care systems that exist in different countries around the world.
- Foster and support innovations to improve the quality of donor human milk through optimized pasteurization or alternative processing techniques. The aim would be to maintain the biological and nutritional quality of human milk while ensuring microbiological safety.
- Foster and support innovations for simplified, low-cost technologies to ensure that safe, quality human milk-banking systems are feasible for low-resource settings.

**Quality management system**

- Include a requirement for hazard analysis critical control points planning as a key strategy in human milk-banking systems to guide the development of rigorous quality control measures specific to the local setting.
- Develop global and regional coordinating bodies to align quality assurance approaches and provide mentorship, oversight and external audit support.

**Data collection and outcome registries**

- Strengthen global indicators to accurately quantify current feeding and lactation practices for small and sick newborns, including human milk intake and actual demand for donor human milk at a global level.
- Integrate global and regional neonatal morbidity and mortality reporting systems with data on neonatal lactation and human milk banking to generate data on the link between feeding and neonatal outcomes.
- Create global and regional tracking systems to link human milk-banking data around the world, creating a communications platform and monitoring tool for assessing and aligning coordinated response to surges in needs and demands (such as natural disasters).

**Breastfeeding mothers**

- Integrate human milk banking into breastfeeding promotion programmes, with the shared goal of prioritizing maternal lactation and using donor human milk only when needed.
- Prioritize the provision of specialized and skilled lactation support as a core component of neonatal care to address the unique needs of mothers of small and sick newborns. Develop training, accreditation and mentorship systems to fill this support gap.
- Strengthen policies and optimize neonatal intensive care to increase mother–infant contact and to enable mother’s own milk to reach her infant, even when separated.

**Ethical issues**

- Conduct a global analysis of commercial human milk banks to comprehensively describe current practices and inform an appropriate response.
- Develop an ethical framework to guide country-specific legislation on the appropriate acquisition and use of donor human milk. The framework would be grounded in the principles of equity and fairness, incorporating protections for milk donors and recipients, and a respect for autonomy and human rights.
- Develop country-specific guidance to overcome impediments to fair access to donor human milk (such as insurance coverage).
- Address global inequities in access to donor human milk by focusing on the expansion and sustainable development of human milk banking in low-resource settings.

**Need for a formalized global coordination body**

- Establish a working group on human milk banking with global representation from different cultural and socioeconomic groups. The group would develop a charter for suitable oversight and communication across human milk banks globally.
- Include expertise on human milk banking in newborn and nutrition policy and technical committees, both globally and regionally to ensure formal engagement and alignment among these groups is achieved. Policy-makers should work with technical experts and health-care teams involved in both neonatal health and neonatal nutrition to ensure their goals are in alignment.
